# Double-Layer Magnetic Nanoparticle-Embedded Silica Particles for Efficient Bio-Separation

**DOI:** 10.1371/journal.pone.0143727

**Published:** 2015-11-24

**Authors:** San Kyeong, Cheolhwan Jeong, Homan Kang, Hong-Jun Cho, Sung-Jun Park, Jin-Kyoung Yang, Sehoon Kim, Hyung-Mo Kim, Bong-Hyun Jun, Yoon-Sik Lee

**Affiliations:** 1 School of Chemical and Biological Engineering, Seoul National University, Seoul, Korea; 2 Nano Systems Institute and Interdisciplinary Program in Nano-Science and Technology, Seoul National University, Seoul, Korea; 3 Center for Theragnosis, Korea Institute of Science and Technology, Seoul, Korea; 4 Department of Bioscience and Biotechnology, Konkuk University, Seoul, Korea; RMIT University, AUSTRALIA

## Abstract

Superparamagnetic Fe_3_O_4_ nanoparticles (NPs) based nanomaterials have been exploited in various biotechnology fields including biomolecule separation. However, slow accumulation of Fe_3_O_4_ NPs by magnets may limit broad applications of Fe_3_O_4_ NP-based nanomaterials. In this study, we report fabrication of Fe_3_O_4_ NPs double-layered silica nanoparticles (DL MNPs) with a silica core and highly packed Fe_3_O_4_ NPs layers. The DL MNPs had a superparamagnetic property and efficient accumulation kinetics under an external magnetic field. Moreover, the magnetic field-exposed DL MNPs show quantitative accumulation, whereas Fe_3_O_4_ NPs single-layered silica nanoparticles (SL MNPs) and silica-coated Fe_3_O_4_ NPs produced a saturated plateau under full recovery of the NPs. DL MNPs are promising nanomaterials with great potential to separate and analyze biomolecules.

## Introduction

Magnetic nanoparticles (MNPs) are of great interest in various biotechnologies, particularly in the field of biomolecule separation [1–3]. Among various MNPs, superparamagnetic Fe_3_O_4_ NPs are promising because they are free from agglomeration by remnant magnetization at room temperature [4–9]. Moreover, they are nontoxic, and the synthetic methods have been well-established [4]. However, bare Fe_3_O_4_ NPs are hampered by several drawbacks to use in biomolecule separation. First, single Fe_3_O_4_ NP itself show very slow accumulation and low separation yield by magnet. Second, Fe_3_O_4_ NPs composed of metal atoms are highly vulnerable to oxidative substances, which would lower the magnetism and dispersion property of NPs [10,11]. Furthermore, still several factors could induce agglomeration in general nanosystems; high surface area to volume ratio of NPs [4,12,13] and van der Waals attractive force [14,15].

Encapsulating a nanostructured template with multiple layers of Fe_3_O_4_ NPs has been considered to overcome these problems. Multiple Fe_3_O_4_ NPs incorporated nanostructures could provide quicker accumulation kinetics under an external magnetic field [16,17]. Silica is one of the desired materials to encapsulate multiple Fe_3_O_4_ NPs because of its chemical inertness, ease of functionalization. Furthermore, being water compatible, silica-encapsulated NPs could be easily exploited to the water-based applications [18]. Various techniques have been reported to synthesize multiple Fe_3_O_4_ NPs-incorporated silica nanostructures [19–22]. However, the NPs should have a well-defined structure and show efficient accumulation kinetics when exposed under a commercial magnet to be successful for biomolecule separation.

In this study, we prepared Fe_3_O_4_ NPs double-layered silica nanoparticles (DL MNPs) with a core/multi-shell structure. The catecholate-iron complexation method was utilized to increase the loading level of Fe_3_O_4_ NPs on the silica NPs. The DL MNPs which contain a large number of Fe_3_O_4_ NPs on the surface of a silica NP core (~200 nm) have monodispersity with strong response for magnetic field with superparamagnetic property. The exploited Fe_3_O_4_ NPs were excellently protected by silica shell from the aforementioned drawbacks. Finally, the capability of DL MNP to separate proteins was demonstrated using ligand-conjugated DL MNPs.

## Experimental Details

### Chemicals and materials

All chemicals were used as-received without further purification. A dispersion of Fe_3_O_4_ NPs (mean diameter, 18 nm, oleate-stabilized in chloroform) was purchased from Ocean Nanotech (Springdale, AR, USA). Polyvinylpyrrolidone (PVP-10K), tetraethyl orthosilicate (TEOS), 3-aminopropyltriethoxysilane (APTS), succinic anhydride, dopamine hydrochloride, biotin, and streptavidin-FITC were purchased from Sigma-Aldrich (St. Louis, MO, USA). Diethyl ether (anhydrous), dimethylformamide (DMF), dichloromethane (DCM), ethanol (EtOH), isopropyl alcohol (IPA), and ammonium hydroxide (28 wt% in water) were obtained from Daejung Chemicals (Daejeon, Korea). Diisopropylethylamine (DIEA) was purchased from Alfa Aesar (Ward Hill, MA, USA). Absolute ethanol (99.5%, HPLC grade) was purchased from Carlo Erba (Milan, Italy). 2-(1*H*-Benzotriazole-1-yl)-1,1,3,3-tetramethyluronium hexafluorophosphate (HBTU) and hydroxybenzotriazole (HOBt) were purchased from Beadtech (Seoul, Korea).

Mouse embryonic fibroblast cell line NIH 3T3, human caucasian prostate adenocarcinoma cell line PC-3 and human glioblastoma astrocytoma cell line U-87 MG were obtained from the Korean Cell Line Bank (Seoul, Korea). All cells were cultured at 37°C with 5% CO_2_ under fully humidified conditions, supplemented with 10% fetal bovine serum (FBS) (Invitrogen, Grand Island, NY, USA), 10 U mL^−1^ of penicillin (Invitrogen, Grand Island, NY, USA), and 10 μg mL^−1^ of streptomycin.

### Synthesis of dopamine-conjugated SiO_2_ NPs

TEOS (1.6 mL) and ammonium hydroxide (4 mL) were added gradually to absolute EtOH (40 mL). The reaction mixture was stirred overnight at 25°C, centrifuged at 7,000 rpm for 15 min, and the resulting SiO_2_ NPs were washed repeatedly with EtOH. Amine groups were introduced to the SiO_2_ NPs (2 mg mL^−1^, 20 mL in EtOH) with APTS (100 μL) and ammonium hydroxide (100 μL) for 18 h. The reaction mixture was centrifuged at 7,000 rpm for 15 min, and the resulting SiO_2_ NPs were washed several times with EtOH, and re-dispersed in DMF. The amine-functionalized SiO_2_ NPs (2 mg mL^−1^, 20 mL in DMF) were reacted with succinic anhydride (40 mg) and DIEA (40 μL). The reaction mixture was stirred for 3 h and washed with DMF several times by centrifugation. The carboxyl group ended- SiO_2_ NPs (2 mg mL^−1^, 20 mL in DMF) were reacted with dopamine hydrochloride (56 mg) and the same equivalent of HOBt, HBTU, and DIEA for 3 h. The dopamine-loaded SiO_2_ NPs were washed several times with DMF by centrifugation (7,000 rpm for 15 min) and redispersed in DMF.

### Ligand exchange of oleate-stabilized Fe_3_O_4_ NPs with PVP

Oleate-stabilized Fe_3_O_4_ NPs (25 mg/mL in 100 μL chloroform) were poured into a DMF/DCM co-solvent (1:1 v/v, 5 mL), and PVP (60 mg) was added. The reaction mixture was heated to 100°C for 18 h, cooled to room temperature, and poured into 10 mL of diethyl ether solution. The solution was centrifuged at 4,500 rpm for 5 min, and the resulting precipitates were redispersed in EtOH.

### Synthesis of SL MNPs and DL MNPs

Dopamine-conjugated SiO_2_ NPs (0.2 mg mL^−1^ DMF, 5 mL in DMF) were poured into a PVP-stabilized Fe_3_O_4_ NPs solution (0.14 mg mL^−1^, 5 mL in EtOH) and sonicated for 1 h. The reaction mixture was centrifuged at 7,000 rpm for 10 min, and washed three times with and dispersed in IPA. TEOS (50 μL) and ammonium hydroxide (125 μL) were added to the Fe_3_O_4_ NPs-immobilized SiO_2_ NP solution (0.2 mg mL^−1^, 5 mL in IPA) and stirred for 18 h. The reaction mixture was centrifuged and washed several times with and dispersed in EtOH. The DL MNPs were synthesized from SL MNPs by repeating the same process.

### Characterization of the NPs

Transmission electron microscopic (TEM) images of NPs were obtained with a JEOL JEM1010 (Tokyo, Japan) for normal use and a JEOL JEM 3010 (Tokyo, Japan) for high resolution imaging analysis, respectively. Hydrodynamic size and the degree of dispersion of the NPs were determined using the dynamic light scattering spectrometer Nanosight (LM10). Fluorescence microscopic images were obtained with a Perkin-Elmer LS55 instrument (Waltham, MA, USA). Field-dependent magnetization of the dried DL MNPs was measured with the Quantum Design PPMS-14 instrument.

### Analysis of NP accumulation rate under an external magnetic field

Three types of NPs (SL MNPs, DL MNPs, and silica-coated Fe_3_O_4_ NPs) were dispersed in EtOH. The NPs were attracted with a magnet (SuperMag Multitube Separator with magnetic strength 1.2 T, Ocean Nanotech), and a 10 μL aliquot of solution was harvested at 0, 1, 2, 3, 5, 10, and 30 min. The concentrations of the initial and harvested NPs were analyzed using Nanosight LM10.

### Stability test of prepared MNPs

A 0.25 mg portion of SL MNPs was added to three kinds of buffer solutions; pH 4.0 potassium hydrogen phthalate buffer (50 mM, 1 mL), pH 7.0 potassium hydrogen phosphate buffer (50 mM, 1 mL), and pH 10 sodium borate buffer (50 mM, 1 mL), respectively. After storage for one week at 25°C, three samples were collected for analysis.

For stability analysis in cell culture medium, a 0.25 mg portion of the SL MNPs was added into cell culture medium; 10% FBS, 10 U mL^−1^ of penicillin, and 10 μg mL^−1^ of streptomycin, and stored for 7 days at 4°C.

### Analysis of biomolecule separation

DL MNPs (0.2 mg mL^−1^, 5 mL in EtOH) were reacted with APTS (50 μL) and ammonium hydroxide (50 μL). The reaction mixture was washed several times with EtOH and re-dispersed in DMF. The resulting amine-functionalized NPs (1 mg mL^−1^, 1 mL in DMF) were reacted with biotin (2.44 mg) and the same equivalent of HBTU, HOBT, and a double equivalent of DIEA for 3 h. The reaction mixture was washed several times with DMF and redispersed in PBS buffer (10 mM, pH 7.4). The biotin-conjugated DL MNPs (1 mg mL^−1^, in 1 mL PBS) were mixed with a streptavidin-FITC conjugate (5 mg mL^−1^, 20 μL) and stirred for 1 h. Then, the mixture was washed three times with PBS buffer.

### Cytotoxicity study

Three types of cell lines, NIH 3T3, PC-3 and U-87 MG, were seeded at 5 × 10^3^ cells in a 96-well microplate, respectively, and DL MNPs of increasing concentration, ranging from 25 to 200 μg, were transferred to the seeded each cells. After incubation for 24 h at 37°C, the cellular toxicity was examined using a simple cellular toxicity kit, cell counting kit-8 (CCK-8) (Dojindo Molecular Tech, Inc, Rockville). The incubated cell medium was clearly removed via a phosphate buffered saline (PBS) washing step, and 10 μ L of the CCK-8 solution containing fresh cell medium were placed into each 96-well microplate. After incubation of the CCK-8-treated samples for 2 h, their absorbance at 450 nm was recorded using a microplate reader.

## Results and Discussion

**Fig 1:**
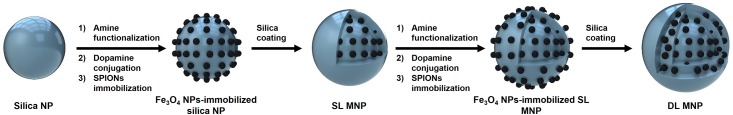
Synthetic scheme for the Fe_3_O_4_ nanoparticles single-layered nanoparticles (SL MNPs) and double-layered nanoparticles (DL MNPs). Fe_3_O_4_ NPs are immobilized to catechol-modified silica NPs; then, SL MNPs are prepared by silica-shell encapsulation. DL MNPs are prepared by repeating the aforementioned method to catechol-modified SL MNPs.

The fabrication flow for the proposed DL MNPs is illustrated in Fig 1. The DL MNPs had a core-shell structure; multiple Fe_3_O_4_ NPs were placed on the shell of the silica core as a laminated double-layer structure. The silica NPs were used as the core template for ease of handling and reproducible preparation. The Fe_3_O_4_ NPs immobilized double-layer was designed to increase the loading level of the MNPs on the silica nanostructure to achieve a strong magnetic property. The surface of the DL MNPs was finally covered with a silica layer for biocompatibility and easy functionalization.

**Fig 2:**
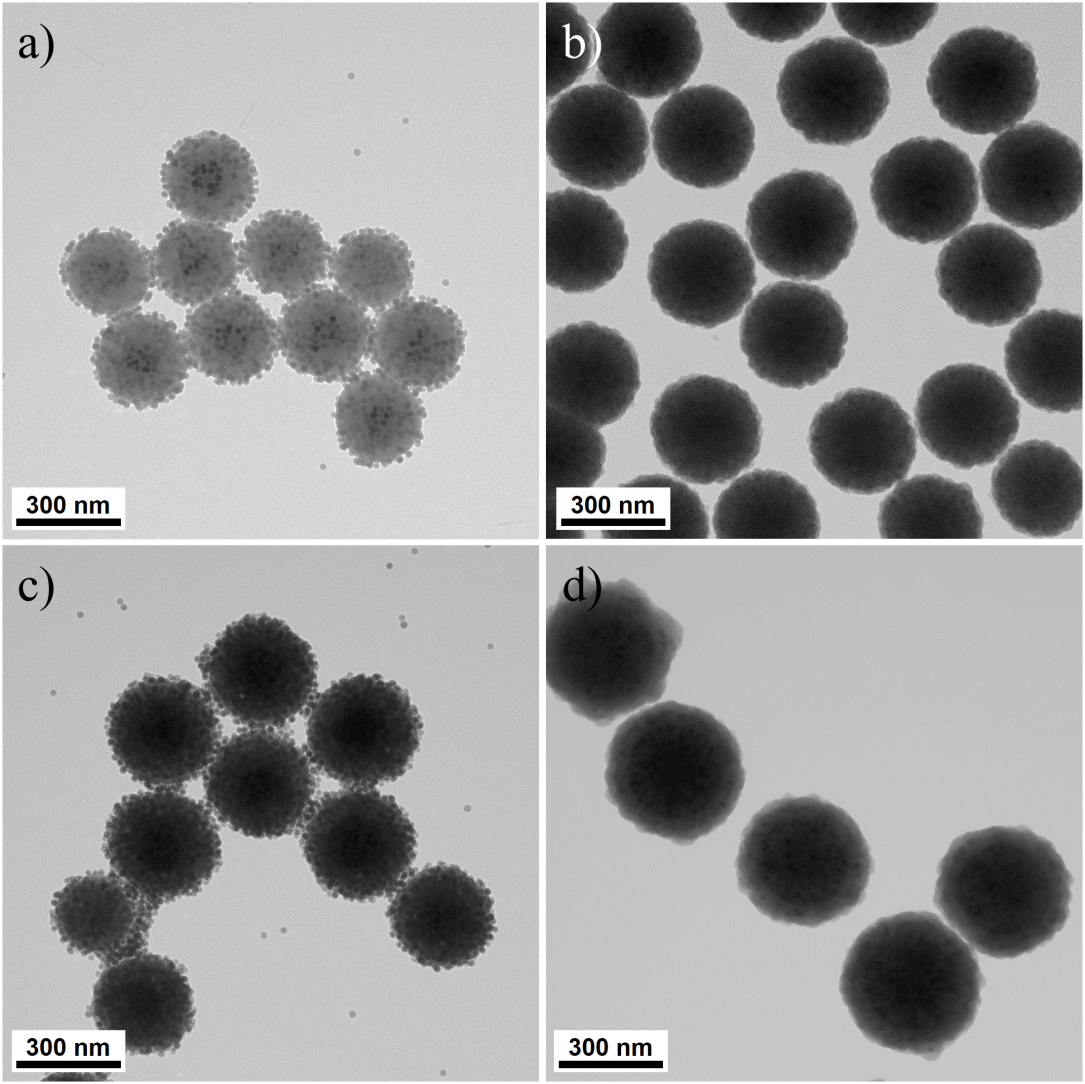
Transmission electron microscopic (TEM) images of the prepared magneticNPs. Images of (a) Fe_3_O_4_ NPs-immobilized SiO_2_ NPs, (b) SL MNPs, (c) Fe_3_O_4_ NPs-immobilized SL MNPs, and (d) DL MNPs.

The DL MNPs were fabricated using the following scheme. First, the core silica NPs were prepared using the Stöber method [23]. The silica NPs were spherical with a diameter of ~200 nm. Then, carboxylic group-ended silica NPs (SiO_2_-COOH NPs) were prepared using a general silica surface modification method with APTS [24] and succinic anhydride [25]. A sidrophore-inspired method was adapted to efficiently immobilize Fe_3_O_4_ NPs on the surface of the silica NPs [26,27]. Dopamine was introduced on the surface of the SiO_2_-COOH NPs by forming an amide bond. The 18 nm-sized amphiphilic PVP-stabilized Fe_3_O_4_ NPs (S1 Fig), which were prepared separately, were immobilized on the dopamine-conjugated SiO_2_ NPs (dop-SiO_2_ NPs) by forming a catecholate-iron complex. As shown in Fig 2a, Fe_3_O_4_ NPs were densely immobilized on the dop-SiO_2_ NP surface (~400 Fe_3_O_4_ NP units/silica core particle). The catecholate-iron complex between the PVP-coated Fe_3_O_4_ NPs and the dop-SiO_2_ NPs maintained their structure after 1 h of sonication. To identify the phase of exploited Fe_3_O_4_ NPs before and after ligand exchange, each of oleate-stabilized Fe_3_O_4_ NPs and the immobilized ones on dopamine-coated SiO_2_ NPs were analyzed using high resolution transmission electron microscope (HR-TEM), respectively. The distance between two adjacent planes of oleate-stabilized Fe_3_O_4_ NP is measured to be 4.9 Å (S2a Fig), corresponding to the typical parameter of (111) plane of the inverse spinel structured Fe_3_O_4_. The immobilized Fe_3_O_4_ NPs onto dopamine-coated SiO_2_ NPs have a lattice parameter of 2.8 Å, which is well matched with (220) plane of Fe_3_O_4_ (S2b Fig). These results demonstrate that Fe_3_O_4_ NPs maintained their structure after the ligand exchange.

The Fe_3_O_4_-immobilized SiO_2_ NPs were washed using additional EtOH to remove residual free Fe_3_O_4_ NPs and then, TEOS was added to form the silica shell. The SL MNPs were encapsulated in a monodispersed manner with a silica shell of ~30 nm thickness, as shown in Fig 2b. The remaining PVP moiety on the NP surface may have affected successful silica coating because its amphiphilic character could enhance affinity between Fe_3_O_4_ NPs and silica [28]. The size of the SL MNPs was ~300 nm, and the number of immobilized Fe_3_O_4_ NPs remained the same after silica coating. DL MNPs were prepared by repeating the aforementioned method. As a result, ~800 units of Fe_3_O_4_ NPs were additionally loaded onto the NPs, as shown Fig 2c. The two-fold higher Fe_3_O_4_ NPs loading on the SL MNPs than the bare dop-SiO2 NPs was the result of an almost two-fold larger surface area.

After introducing additional Fe_3_O_4_ NP layers and a silica shell (~30 nm), the size of the DL MNPs was ~400 nm, as shown in Fig 2d. The prepared NPs were uniform in size and no aggregation of MNPs was detected. In addition, ~1,200 Fe_3_O_4_ NP units were successfully packed into a single nanostructure due to the high affinity between the catechol moiety and iron oxide as well as the double-layered structure.

**Fig 3:**
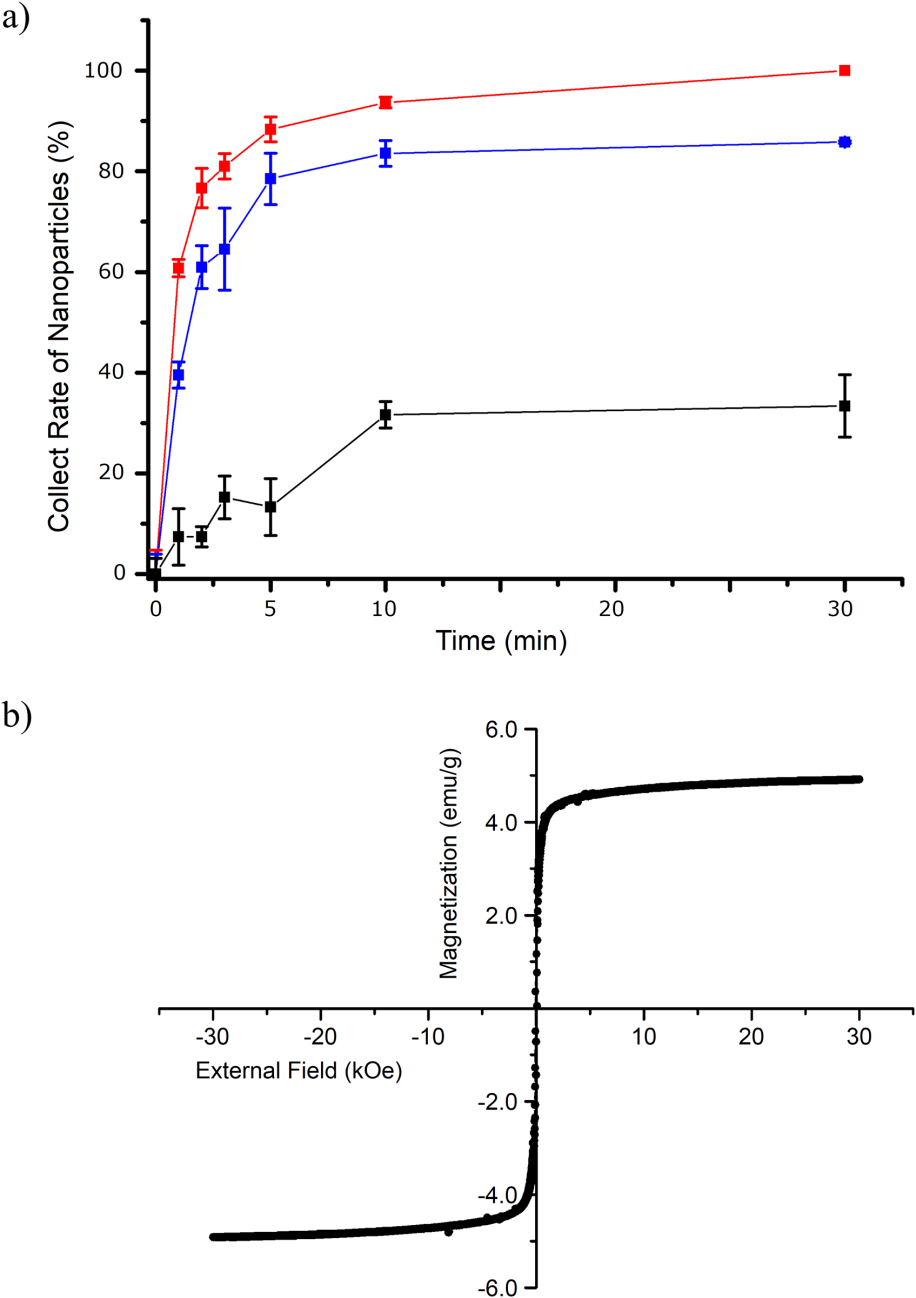
Magnetic properties of the prepared magnetic NPs. a) Accumulation profiles for the DL MNPs (red), SL MNPs (blue), and the silica-coated Fe_3_O_4_ NPs (black). b) Hysteresis loop of the DL MNPs.

We investigated the magnetic properties of the SL MNPs and DL MNPs. There are enormous amounts of studies of the MNPs-exploited biomolecule separation. Many successful results have been reported for target cell separation, gaining more than 70–90% separation efficiency [17,29,30]. However, in the case of cell separation, high separation efficiency could be obtained even though magnetic strength of the exploited MNPs is low, because large numbers of MNPs could be attached to a single cell. On the other hand, to obtain high efficiency of separating smaller biomolecules, magnetic strength of single MNP itself can be a critical issue. First, we analyzed their accumulation kinetics using an external magnetic field. Samples of SL MNPs, DL MNPs, and silica-coated Fe_3_O_4_ NPs in a 1.5 mL microtube were separated with a NdFeB magnet. The silica-coated single Fe_3_O_4_ NPs (S3 Fig) were prepared using our method reported previously [31]. A small aliquot of the NP suspension was harvested, and the concentration of MNPs was measured at selected time intervals (1, 2, 3, 5, 10, and 30 min after placing the magnet). The accumulation rate was obtained by subtracting the measured concentration of the MNPs from the initial concentration. As shown in Fig 3a, DL MNPs were collected more efficiently than silica-coated Fe_3_O_4_ NPs or SL MNPs. Although the SL MNPs showed a better accumulation profile compared to that of the silica-coated Fe_3_O_4_ NPs, both NPs produced a saturated plateau underneath full recovery. The silica surface charge of the prepared MNPs could cause a charge-charge repulsion, which might lead to incomplete accumulation of NPs. However, all DL MNPs accumulated quantitatively in 30 min, indicating that the DL MNPs contained a sufficient number of Fe_3_O_4_ NPs to overcome the repulsive interactions of the silica-surfaced NPs. The field-dependent magnetism of the dried DL MNPs at 300 K exhibited superparamagnetic characteristics (Fig 3b), with a saturated magnetization of 5.0 emu/g. In addition, the immobilized Fe_3_O_4_ NPs onto the MNP retained their magnetic property for 2 month at room temperature (S4 Fig). Although the magnetization value per weight was lower than 18 nm MNPs (60.0 emu/g), each DL MNP unit had a 500-fold stronger magnetic property than that of bare 18 nm MNPs. Details of the calculations and assumptions are described in S1 File [32].

To confirm the stability of the prepared MNPs, the SL MNPs were stored at three kinds of buffer solution of pH 4, 7 and 10 for 7 days, respectively. The SL MNPs maintained their original structure as well as the dispersion property and none of Fe_3_O_4_ NPs was leached out under acidic (pH 4, S5a Fig) and neutral condition (pH 7, S5b Fig). On the other hand, the SiO_2_ layer surface of SL MNPs was slightly degraded under basic buffer condition (pH 10, S5c Fig), although Fe_3_O_4_ NPs leaching was not found. Since most of silica-based materials have been successfully performed under pH 6~8 [33–36], our MNPs might be suitable for common bio-application.

**Fig 4:**
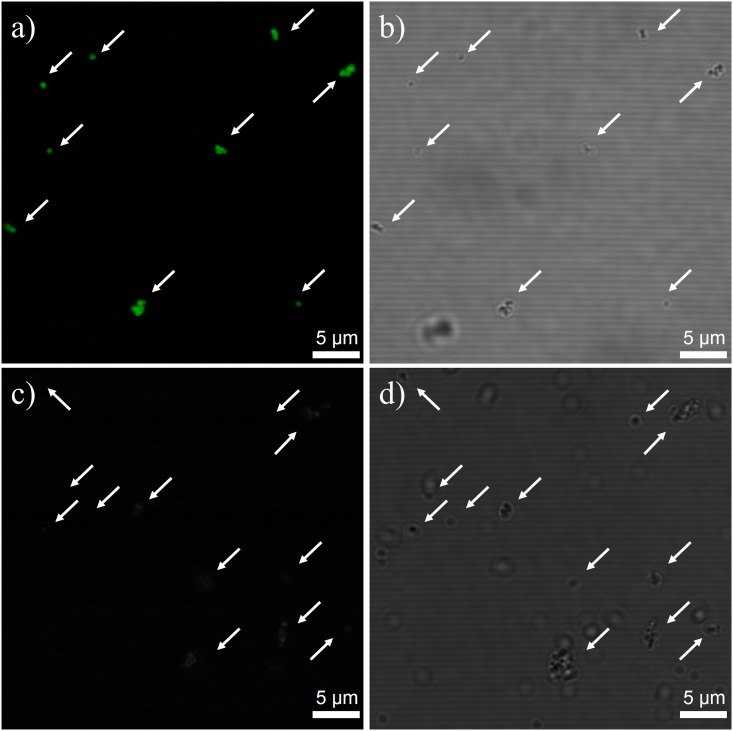
FITC-streptavidin separation using biotin-conjugated DL MNPs and bare DL MNPs. Fluorescence and optical microscopic images of (a, b) biotin-conjugated DL MNPs and (c, d) bare DL MNPs after incubation with FITC-streptavidin and magnet-induced separation. Arrows indicate the detected NPs.

Next, streptavidin was separated to demonstrate that the NPs could be used to separate proteins with a magnet. Biotin-conjugated DL MNPs and bare DL MNPs were incubated with FITC-streptavidin in a microtube. Biotin conjugated easily to the NPs through a typical amine functionalization onto the silica surface followed by amide coupling. A magnet was placed close to one side of the two sample tubes for 30 min. Thereafter, the streptavidin captured MNP suspension was removed, and the captured MNP portion accumulated by the magnet was resuspended in fresh PBS buffer. The resuspended samples were analyzed under a confocal-optical microscope. All biotin-conjugated DL MNPs observed under the optical microscope illuminated green fluorescence originating from FITC-streptavidin, indicating that streptavidin was clearly separated out using the biotin-conjugated DL MNPs (Fig 4a and 4b). No green fluorescence of FITC-streptavidin was observed from the bare DL MNPs, as shown in Fig 4c and 4d. In addition, these MNPs maintained their hydrodynamic size for 7 days in cell culture medium (S6 Fig), and did not show significant cytotoxic activity, based on CCK-8 assay results (S7 Fig). These results clearly demonstrate the great potential of DL MNPs for biomolecule separations.

## Conclusion

In summary, core/multi-shell type silica-coated magnetic NPs were synthesized. We utilized a surface modification method based on the catecholate-iron complex and fabricated a double-layered structure to immobilize a large number of Fe_3_O_4_ NPs on silica NPs. As a result, ~1,200 Fe_3_O_4_ NPs-embedded silica NPs were prepared, and the resulting NPs were highly uniform in size, and exhibited a superparamagnetic property. They were quantitatively collected using a magnet in a short period of time. Moreover, FITC-streptavidin was effectively separated using biotin-conjugated DL MNPs under an external magnetic field, showing that the DL MNPs have great potential to be exploited for biomolecule separation.

## Supporting Information

S1 Fig TEM images of polyvinylpyrrolidone-stabilized Fe_3_O_4_ NPs. (TIF)

S2 Fig Lattice fringes of Fe_3_O_4_ NPs before and after the ligand exchange. HR-TEM images of (a) oleate-stabilized Fe_3_O_4_ NPs and (b) immobilized Fe_3_O_4_ NPs onto the surface of dopamine-conjugated SiO_2_ NPs. Arrows indicate the distance between two adjacent planes.
(TIF)

S3 Fig TEM image of silica-coated Fe_3_O_4_ NPs. (TIF)

S4 Fig Magnetic properties of the prepared MNPs under long term storage. Hysteresis loop for SL MNPs which were, a) newly synthesized, and b) stored for 2 months, respectively.
(TIF)

S5 Fig Stability test of SL MNPs under various pH conditions. TEM images of SL MNPs after storage for 7 days at (a, top and bottom) pH 4 potassium hydrogen phthalate buffer, (b, top and bottom) pH 7 potassium hydrogen phosphate buffer, and (c, top and bottom) pH 10 sodium borate buffer, respectively.
(TIF)

S6 Fig Stability analysis of SL MNPs in cell culture medium. Hydrodynamic radius of SL MNPs after storage for 7 days at (a) pH 7 potassium hydrogen phosphate buffer and (b) cell culture medium; 10% fetal bovine serum (FBS), 10 U mL^−1^ of penicillin, and 10 μg mL^−1^ of streptomycin.
(TIF)

S7 Fig CCK-8 assay of DL MNPs. The data were obtained after subtracting the measured intensity of control group, i.e. DL MNPs-containing cell medium.
(TIF)

S1 File Calculation of magnetic force of a DL MNP [emu/unit]. (PDF)

S2 File Quality report data of hydrodynamics radius for SL MNPs in pH 7 buffer. (PDF)

S3 File Quality report data of hydrodynamic radius for SL MNPs in cell culture medium. (PDF)
