# Supplementary figures and images for: Double-Layer Magnetic Nanoparticle-Embedded Silica Particles for Efficient Bio-Separation
Source: PLoS One. 2015 Nov 24;10(11):e0143727. doi: 10.1371/journal.pone.0143727 (PMC4658053; doi:10.1371/journal.pone.0143727)

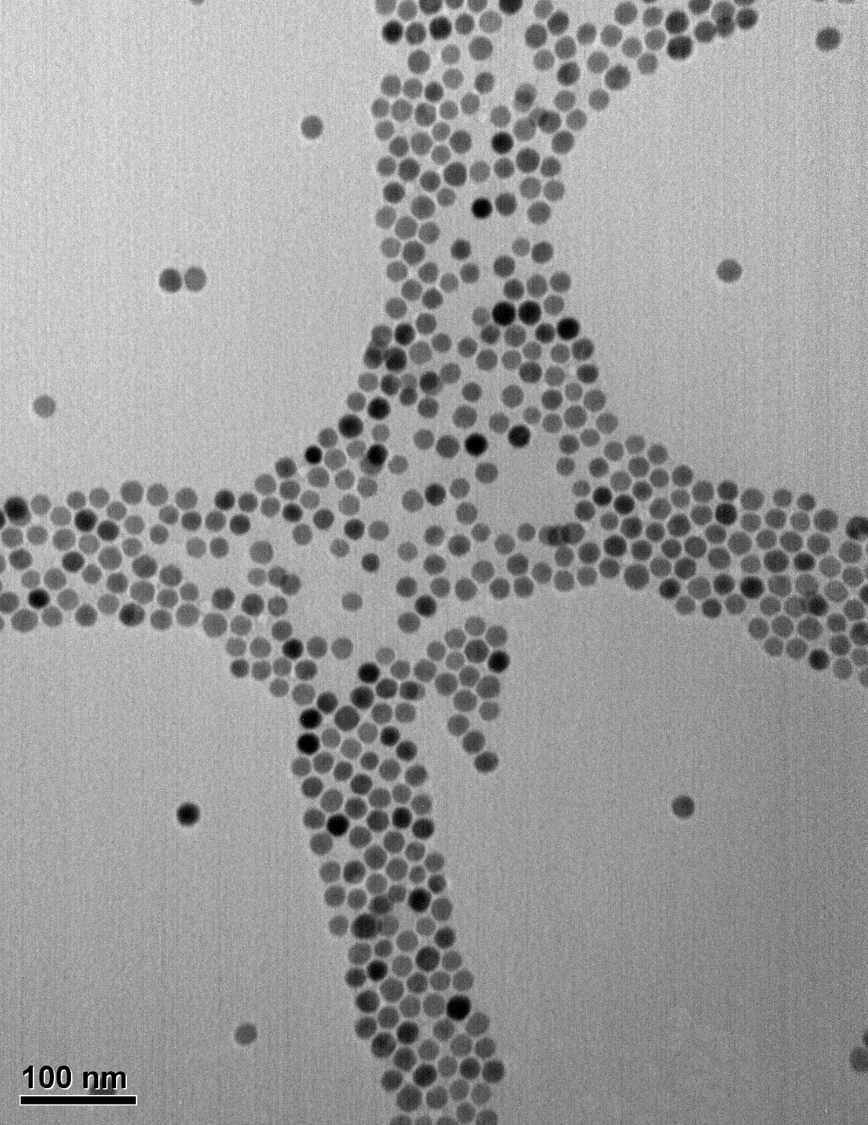

Supplement: S1 Fig — (TIF) [file pone.0143727.s001.tif]

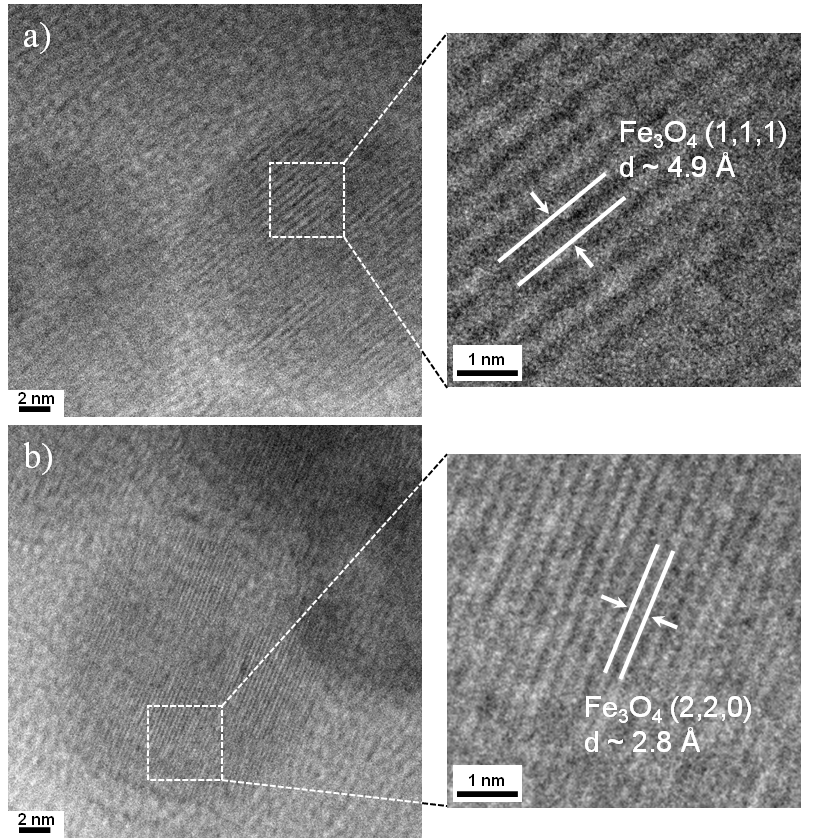

Supplement: S2 Fig — HR-TEM images of (a) oleate-stabilized Fe3O4 NPs and (b) immobilized Fe3O4 NPs onto the surface of dopamine-conjugated SiO2 NPs. Arrows indicate the distance between two adjacent planes. (TIF) [file pone.0143727.s002.tif]

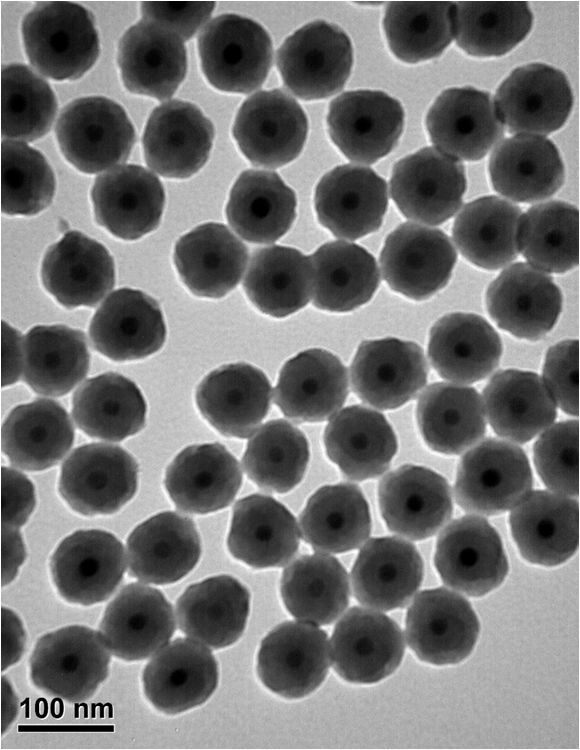

Supplement: S3 Fig — (TIF) [file pone.0143727.s003.tif]

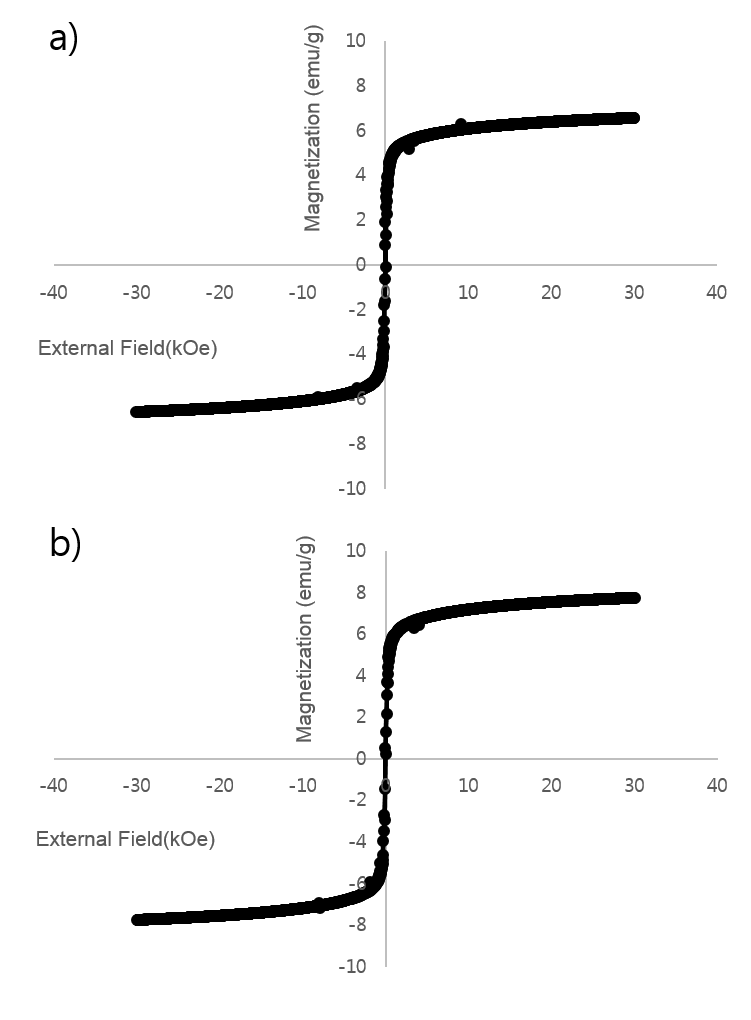

Supplement: S4 Fig — Hysteresis loop for SL MNPs which were, a) newly synthesized, and b) stored for 2 months, respectively. (TIF) [file pone.0143727.s004.tif]

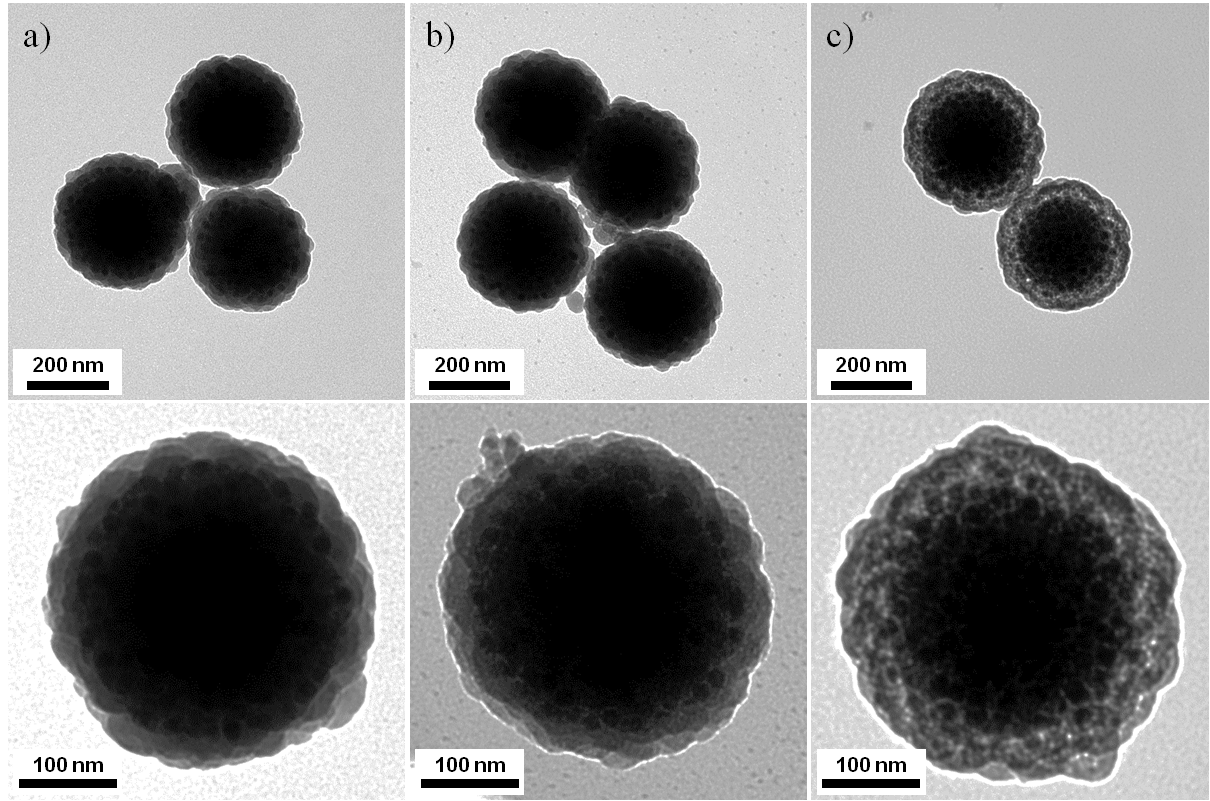

Supplement: S5 Fig — TEM images of SL MNPs after storage for 7 days at (a, top and bottom) pH 4 potassium hydrogen phthalate buffer, (b, top and bottom) pH 7 potassium hydrogen phosphate buffer, and (c, top and bottom) pH 10 sodium borate buffer, respectively. (TIF) [file pone.0143727.s005.tif]

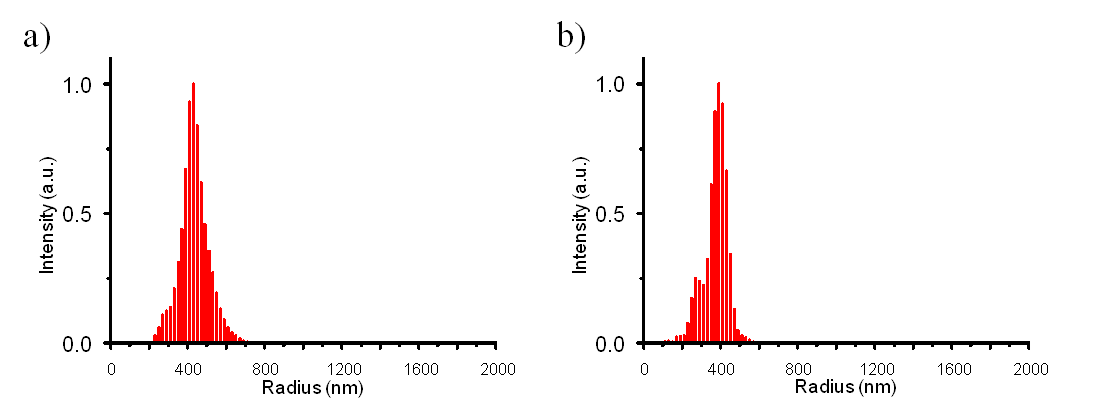

Supplement: S6 Fig — Hydrodynamic radius of SL MNPs after storage for 7 days at (a) pH 7 potassium hydrogen phosphate buffer and (b) cell culture medium; 10% fetal bovine serum (FBS), 10 U mL−1 of penicillin, and 10 μg mL−1 of streptomycin. (TIF) [file pone.0143727.s006.tif]

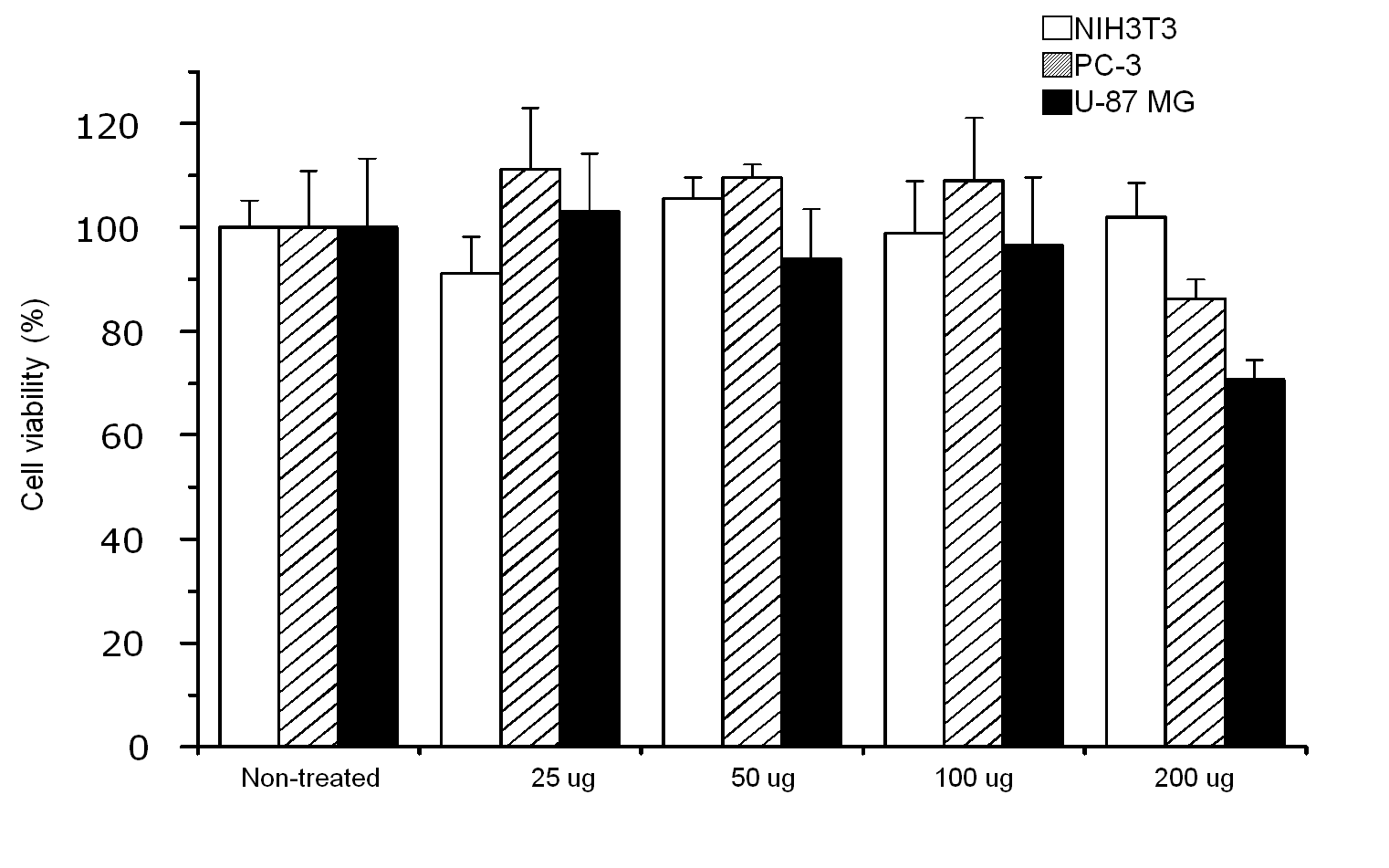

Supplement: S7 Fig — The data were obtained after subtracting the measured intensity of control group, i.e. DL MNPs-containing cell medium. (TIF) [file pone.0143727.s007.tif]
