# Supplementary material for: Double-Layer Magnetic Nanoparticle-Embedded Silica Particles for Efficient Bio-Separation
Source: PLoS One. 2015 Nov 24;10(11):e0143727. doi: 10.1371/journal.pone.0143727 (PMC4658053; doi:10.1371/journal.pone.0143727)
Supplement: S1 File — (PDF) [file pone.0143727.s008.pdf]

Calculation of magnetic force of a single DL MNP [emu/unit]

(a) Magnetic force of a single DL MNP [emu/unit]

= Magnetic force of DL MNPs [emu/g] × weight of a single DL MNP [ng/unit]

= 5.0 [emu/g] × (9.7×10^{-5}) [ng/unit]

= 4.9×10^{-13} [emu/unit]

- The magnetic force of the DL MNPs was 5.0 emu/g
- Weight of 1 DL MNP unit

  = (Volume of 400 nm DL MNP [nm^3] – volume of 18 nm Fe_3O_4 NP [nm^3/unit] × 1200 [unit]) × density of SiO_2 (2.65 g/cm^3) + volume of 18 nm Fe_3O_4 NP [nm^3/unit] × 1200 [unit] × density of Fe_3O_4 (5.0 g/cm^3)

  = 9.7×10^{-5} [ng]

(b) Magnetic force of a single Fe_3O_4 NP [emu/unit]

= Magnetic force of 18 nm Fe_3O_4 NP [emu/g] × weight of single Fe_3O_4 NP [g/unit]

= 60.0 [emu/g] × 1.5×10^{-8} [ng/unit]

= 9.0×10^{-16} [emu/unit]

- The magnetic force of the Fe_3O_4 NPs was 60.0 emu/g (measured by SQUID)
- Weight of a Fe_3O_4 NP unit

  = Volume of 18 nm Fe_3O_4 NP [nm^3/unit] × density of Fe_3O_4 (5.0 g/cm^3)

  = 1.5×10^{-8} [ng]

(c) Magnetic force of a single DL MNP [emu/unit]/magnetic force of a single Fe_3O_4 NP [emu/unit]

= 540

For these calculations, we assumed that:

1. all DL MNPs were completely spherical in shape, with the same size (r = 200 nm, containing magnetite with r = 9 nm)
2. DL MNPs consisted of only silica and Fe_3O_4 NPs. We also assumed that the Fe_3O_4 NPs were composed of only Fe_3O_4.
3. Density of Fe_3O_4 (5.0 g/cm^3) and that of SiO_2 (2.65 g/cm^3)

Reference Ovsyannikov, S. V., Shchennikov, V. V., Todo, S. & Uwatoko, Y. A new crossover in Fe_3O_4 magnetite under pressure near 6 GPa: modification to ‘ideal’ inverse cubic spinel?. *J. Phys.: Condens. Matter* **20**, 172201 (6 pp.) (2008).
