# Supplementary material for: Double-Layer Magnetic Nanoparticle-Embedded Silica Particles for Efficient Bio-Separation
Source: PLoS One. 2015 Nov 24;10(11):e0143727. doi: 10.1371/journal.pone.0143727 (PMC4658053; doi:10.1371/journal.pone.0143727)
Supplement: S2 File — (PDF) [file pone.0143727.s009.pdf]

NANOSIGHT

ANALYSIS REPORT

Nanoparticle Tracking Analysis (NTA) Version 2.3 Build 0033

Sample:
Date/Time of Capture: 1 July 2015 12:31
Video File: san132-2.avi analysis no: 040
Operator: KKH
Comments:

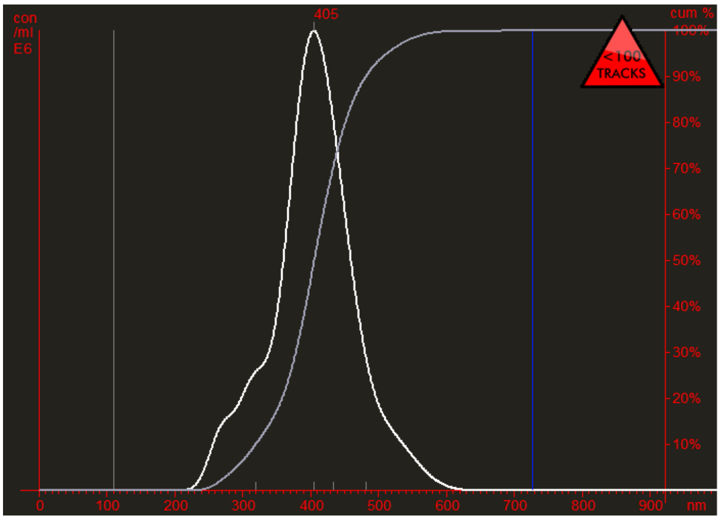

Particle Size / Concentration

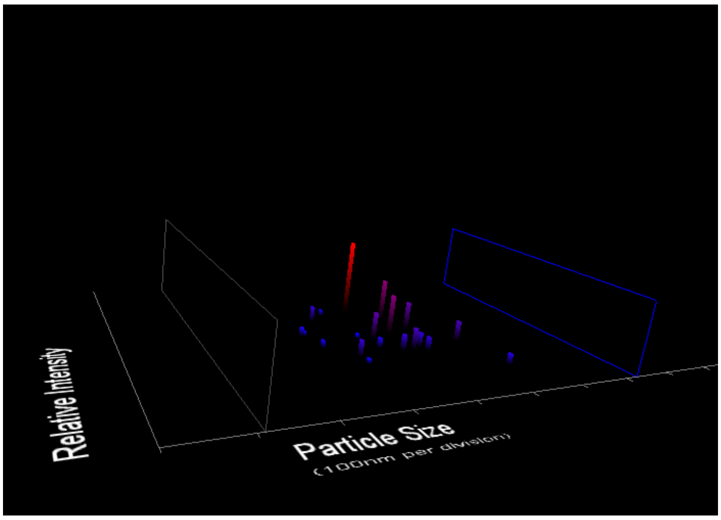

Particle Size / Relative Intensity 3D plot

Bin Centre (nm)	Concentration (E6 particles/ml)	Percentile Undersize (%)
10	0.000	0.000
30	0.000	0.000
50	0.000	0.000
70	0.000	0.000
90	0.000	0.000
110	0.000	0.000
130	0.000	0.000
150	0.000	0.000
170	0.000	0.000
190	0.000	0.000
210	0.009	0.013
230	0.148	0.220
250	0.785	1.317
270	1.598	3.548
290	2.015	6.361
310	2.635	10.040
330	3.100	14.369
350	4.264	20.322
370	7.186	30.355
390	10.205	44.604
410	11.051	60.034
430	9.558	73.379
450	7.013	83.171
470	4.527	89.492
490	2.755	93.339
510	1.804	95.857
530	1.294	97.665
550	0.873	98.884
570	0.488	99.565
590	0.214	99.865
610	0.073	99.967
630	0.019	99.994
650	0.004	99.999
670	0.001	100.000
690	0.000	100.000

Bin Centre (nm)	Concentration (E6 particles/ml)	Percentile Undersize (%)
710	0.000	100.000
730	0.000	100.000
750	0.000	100.000
770	0.000	100.000
790	0.000	100.000
810	0.000	100.000
830	0.000	100.000
850	0.000	100.000
870	0.000	100.000
890	0.000	100.000
910	0.000	100.000
930	0.000	100.000
950	0.000	100.000
970	0.000	100.000
990	0.000	100.000
1000-2000	0.000	100.000

Results

Mean: **406 nm**
Mode: **405 nm**
SD: **57 nm**
D10: **319 nm**
D50: **406 nm**
D90: **482 nm**
User Lines: **110 nm, 727 nm**
Concentration: **0.72 E8 particles/ml**
Completed Tracks: **23**

Measurement Conditions

Temperature: 23.00 ºC
Viscosity: 0.93 cP
Frames Per Second: 30.00
Measurement Time: 60 of 60 s
Drift Velocity: 361 nm/s
Camera Shutter: 30 ms

Analysis Conditions

Blur: 3x3
Detection Threshold: 10 Multi
Min Track Length: 49
Min Expected Size: Auto
