# Supplementary material for: Double-Layer Magnetic Nanoparticle-Embedded Silica Particles for Efficient Bio-Separation
Source: PLoS One. 2015 Nov 24;10(11):e0143727. doi: 10.1371/journal.pone.0143727 (PMC4658053; doi:10.1371/journal.pone.0143727)
Supplement: S3 File — (PDF) [file pone.0143727.s010.pdf]

NANOSIGHT

ANALYSIS REPORT

Nanoparticle Tracking Analysis (NTA) Version 2.3 Build 0033

Sample:
Date/Time of Capture:
Video File: san143.avi analysis no: 001
Operator:
Comments:

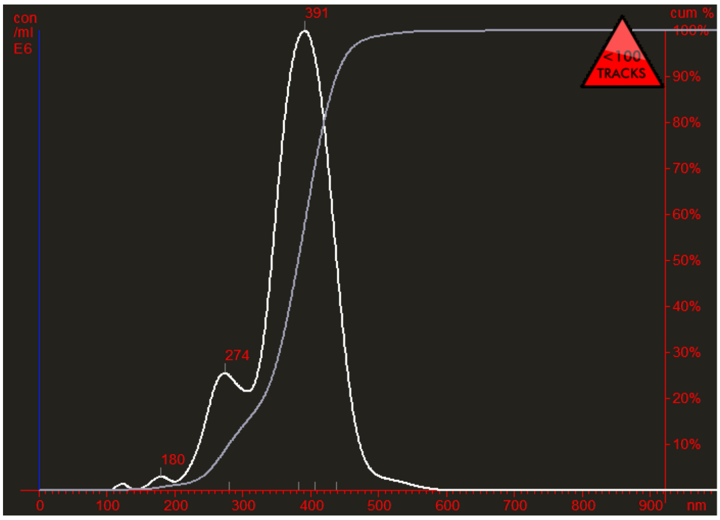

Particle Size / Concentration

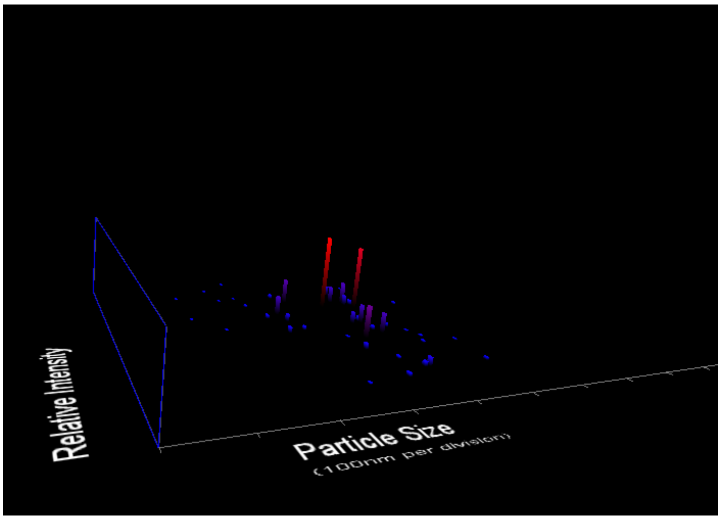

Particle Size / Relative Intensity 3D plot

Bin Centre (nm)	Concentration (E6 particles/ml)	Percentile Undersize (%)
10	0.000	0.000
30	0.000	0.000
50	0.000	0.000
70	0.000	0.000
90	0.000	0.001
110	0.040	0.093
130	0.072	0.262
150	0.040	0.355
170	0.167	0.745
190	0.181	1.168
210	0.202	1.640
230	0.537	2.895
250	1.219	5.743
270	1.765	9.865
290	1.678	13.784
310	1.571	17.455
330	2.282	22.786
350	4.307	32.846
370	6.299	47.560
390	7.055	64.039
410	6.522	79.272
430	4.691	90.229
450	2.424	95.891
470	0.924	98.049
490	0.343	98.849
510	0.196	99.307
530	0.144	99.643
550	0.091	99.855
570	0.043	99.955
590	0.015	99.990
610	0.004	99.998
630	0.001	100.000
650	0.000	100.000
670	0.000	100.000
690	0.000	100.000

Bin Centre (nm)	Concentration (E6 particles/ml)	Percentile Undersize (%)
710	0.000	100.000
730	0.000	100.000
750	0.000	100.000
770	0.000	100.000
790	0.000	100.000
810	0.000	100.000
830	0.000	100.000
850	0.000	100.000
870	0.000	100.000
890	0.000	100.000
910	0.000	100.000
930	0.000	100.000
950	0.000	100.000
970	0.000	100.000
990	0.000	100.000
1000-2000	0.000	100.000

Results

**Mean:**	**374 nm**
**Mode:**	**391 nm**
**SD:**	**57 nm**
**D10:**	**280 nm**
**D50:**	**383 nm**
**D90:**	**439 nm**
**User Lines:**	**0 nm, 0 nm**
**Concentration:**	**0.43 E8 particles/ml**
**Completed Tracks:**	**46**

Measurement Conditions

Temperature:	22.90 °C
Viscosity:	0.93 cP
Frames Per Second:	30.00
Measurement Time:	60 of 60 s
Drift Velocity:	1025 nm/s
Camera Shutter:	14 ms

Analysis Conditions

Blur:	Auto
Detection Threshold:	10 Multi
Min Track Length:	Auto
Min Expected Size:	Auto
